# Role for MMP-9 in stress-induced downregulation of nectin-3 in hippocampal CA1 and associated behavioural alterations

**DOI:** 10.1038/ncomms5995

**Published:** 2014-09-18

**Authors:** Michael A. van der Kooij, Martina Fantin, Emilia Rejmak, Jocelyn Grosse, Olivia Zanoletti, Celine Fournier, Krishnendu Ganguly, Katarzyna Kalita, Leszek Kaczmarek, Carmen Sandi

**Affiliations:** 1 Laboratory of Behavioral Genetics, Brain Mind Institute, School of Life Sciences, École Polytechnique Fédérale de Lausanne, EPFL, Lausanne 1015, Switzerland; 2 Laboratory of Neurobiology, Nencki Institute of Experimental Biology, Polish Academy of Sciences, 3 Pasteur Street 02-093 Warsaw, Poland

## Abstract

Chronic stress is a risk factor for the development of psychopathologies characterized by cognitive dysfunction and deregulated social behaviours. Emerging evidence suggests a role for cell adhesion molecules, including nectin-3, in the mechanisms that underlie the behavioural effects of stress. We tested the hypothesis that proteolytic processing of nectins by matrix metalloproteinases (MMPs), an enzyme family that degrades numerous substrates, including cell adhesion molecules, is involved in hippocampal effects induced by chronic restraint stress. A reduction in nectin-3 in the perisynaptic CA1, but not in the CA3, compartment is observed following chronic stress and is implicated in the effects of stress in social exploration, social recognition and a CA1-dependent cognitive task. Increased MMP-9-related gelatinase activity, involving *N*-methyl-D-aspartate receptor, is specifically found in the CA1 and involved in nectin-3 cleavage and chronic stress-induced social and cognitive alterations. Thus, MMP-9 proteolytic processing emerges as an important mediator of stress effects in brain function and behaviour.

## Abstract (web summary)

A role for cell adhesion molecules like nectin-3 in the mechanisms that underlie the behavioural effects of stress have been previously described. Here the authors show that the metalloproteinase matrix metalloproteinase-9 is involved in proteolysis of nectin-3 and in mediating stress induced behavioural effects in rats.

## Graphical abstract

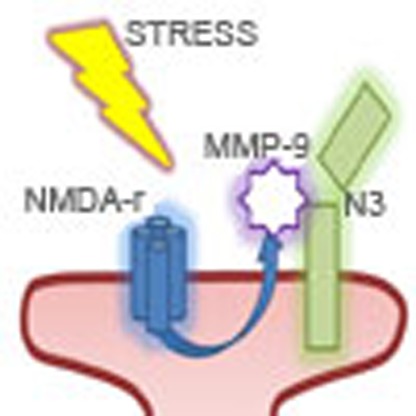

Chronic exposure to stress is an important trigger factor for the development of neuropsychiatric disorders^1^,^2^,^3^. The hippocampus is one of the brain regions that displaying substantial vulnerability to chronic stress^1^, displaying marked structural changes in dendritic complexity^4^ and spine volume and number^5^. Some of these effects were shown to involve the action of excitatory glutamatergic transmission^6^ and to be linked to impairments in learning and memory^7^ and social behaviours^8^. However, the molecular mechanisms involved in the detrimental effects of chronic stress in hippocampus-dependent behaviours are not well understood.

Proteolytic processing in the perisynaptic microenvironment is a central mechanism of neural plasticity that is mainly regulated by various families of proteases, such as matrix metalloproteinases (MMPs) and serine proteases^9^. Evidence was shown for the involvement of serine proteases in stress-induced plasticity and behavioural changes in different brain regions including the hippocampus^10^,^11^,^12^. However, despite mounting evidence critically implicating MMPs as important regulators of structural and functional plasticity^13^, there is no information regarding the potential role of MMPs in stress-induced adaptations.

MMPs are a family of proteases that critically influence cellular behaviour through targeted degradation or proteolytic processing of various extracellular matrix molecules. Although they play key roles in many physiological processes, aberrant MMP expression or function has also been implicated in the molecular mechanisms underlying certain neuropathologies^14^,^15^. The MMP subfamily of gelatinases––the most prominent being MMP-2 and MMP-9—is to date the most studied. These gelatinases have been critically implicated in synaptic circuit remodelling—including structural changes in dendritic spines and axon/dendrite structures—both in health and disease^11^,^16^. Although constitutive levels of these gelatinases in the adult brain are low, MMP-9 expression in the adult rodent hippocampus is particularly increased^17^,^18^ and further enhanced following learning^19^ or synaptic potentiation^20^. MMP-9 is concentrated throughout the synaptic neuropile in discrete puncta that co-localize with presynaptic and postsynaptic markers^17^,^18^ and are particularly abundant in focal gelatinolytic puncta that localize with glutamatergic markers^18^. Whereas focal and transient activation of MMP-9 has been critically implicated in synaptic plasticity underlying cognitive processes, abnormal MMP-9 activity can contribute to cognitive dysfunction^13^. Interestingly, MMP-9 has been found to be elevated in macrophage cells from depressed patients, especially in combination with high levels of stress^21^. However, whether these gelatinases are involved in the effects of chronic stress in brain function and cognition is not yet known.

Importantly, degradation of cell adhesion molecules is one of the main mechanisms whereby MMPs were shown to affect neural plasticity^9^,^22^. Changes in cell adhesion molecules expression are emerging as key players in stress-induced neuronal and cognitive effects^23^. Initially, the neural cell adhesion molecule (NCAM), a molecule that is broadly expressed throughout the cell membrane, was implicated in stress effects in brain and cognition^21^. More recently, a key role has been suggested for cell adhesion molecules with a more restricted location to the (peri)synaptic environment^8^,^24^,^25^. Among them, nectin-3 was found downregulated in the hippocampus following chronic stress^24^ and to be implicated in the effects of acute stress in memory and structural plasticity^25^. However, it is not known whether changes in nectin expression are involved in chronic stress-induced alteration in hippocampus-dependent behaviours and if proteolytic processing plays a role in nectin modulation.

Nectins are Ca^2+^-independent, Ig-like transmembrane proteins mostly found at the perisynaptic regions^26^. In the hippocampus, nectin-1 and nectin-3 are predominantly expressed pre- and post-synaptically, respectively^27^,^28^, with nectin-1–nectin-3 interactions playing a critical role in selective axo-dendritic adhesion^29^. Nectins exert their actions through their association with the actin cytoskeleton via afadin and the recruitment of cadherins^29^,^30^.

Degradation of cell adhesion molecules can occur through proteolytic shedding of the extracellular N-terminal domains and subsequent cleavage of the intracellular domain^9^,^31^. Nectin-1 has been shown to undergo ectodomain shedding by different secretases involving post-synaptic NMDA (*N*-methyl-D-aspartate) receptor activation^32^,^33^. Although cell culture experiments indicate that nectin-3 also undergoes multiple cleavage events^32^, the molecular players involved in nectin-3 proteolytic processing are still unknown. Recent evidence pointed towards MMPs as potential regulators of nectin processing^34^.

Therefore, we aimed here at investigating whether MMP gelatinase activity would be involved in the functional effects of chronic stress. Given the recent evidence pointing at the involvement of nectin-3 in stress actions^24^,^25^, we targeted MMP as a potential regulator. As chronic stress affects different synaptic features in the hippocampal CA3 (ref. 35) and CA1 (ref. 5) regions, we hypothesized that molecular changes will show hippocampal region specificity. We selected the 21-day chronic restraint stress, for its well-characterized effects in hippocampal structure and function^1^,^5^,^36^,^37^. We focused on social behaviours (that is, social exploration, social memory and aggressive behaviours) that are known to be altered by this stress model^8^ and that depend on hippocampal integrity^38^. Our results highlight an important role for MMP-9 activity in stress-induced hippocampus-dependent adaptations. More precisely, MMP-9 activity is implicated in a reduction in the perisynaptic nectin-3 expression specifically in the CA1, but not the CA3 region. Moreover, we demonstrate the involvement of MMP-9 in chronic stress-induced alterations in social behaviours (including reduced sociability and social memory) and CA1-mediated cognition.

## Results

### Altered social behaviour and reduced CA1 nectin-3 after chronic stress

Exposure to chronic social defeat^24^ or to early life stress^25^ in mice was shown to induce a reduction in nectin-3 expression in CA3 region. As we aimed at investigating whether MMP gelatinase activity is involved in the hippocampal regulation of nectin expression in the chronic restraint stress model in rats, we first assessed whether restraint stress leads to changes in nectin-1 and/or nectin-3 protein expression in the hippocampal CA1 and/or CA3. We also aimed at establishing whether any putative changes in the expression of these molecules would occur in parallel with alterations in social behaviours that we have recently reported using this stress protocol^8^.

**Figure 1:**
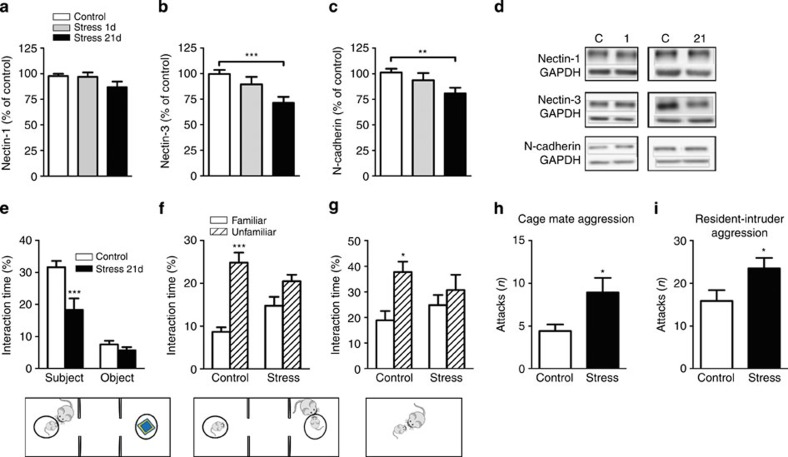
Chronic restraint stress reduces nectin-3 and alters behaviour. (**a**) Chronic restraint stress showed a trend to reduce nectin-1 (F_2,61_=2.499, *P*=0.09, control *n*=32, stress 1 day *n*=13, stress 21 days *n*=17), whereas chronic stress (**b**) significantly reduced nectin-3 (F_2,51_=7.706, *P*=0.0012, control=24) and (**c**) N-Cadherin (F_2,58_=4.465, *P*=0.0159, control *n*=23, stress 1 day *n*=13, stress 21 days *n*=23). (**d**) Representative western blots for CA1 synaptoneurosomes showing nectin-1, nectin-3 and n-cadherin (C=control, 1 and 21 indicates restraint stress for 1 and 21 days, respectively). (**e**) Rats subjected to 21 days of chronic restraint stress spent less time investigating an unfamiliar juvenile (stress effect: F_1,15_=21.1, *P*=0.0003, control *n*=11, stress 21 days *n*=6). (**f**,**g**) Chronic restraint stress impaired social recognition in the three-chambered apparatus and in a freely moving paradigm (three-chambered apparatus: F_1,12_=34.27, *P*<0.001, *n*=7 per group; freely moving: F_1,10_=7.764, *P*=0.0192, *n*=6 per group). (**h**,**i**) Chronic stress enhanced aggressive behaviour towards the cagemate after release from the restrainer and against an unfamiliar male in the resident-intruder paradigm (cagemate: *t*_22_=2.385, *P*=0.0261, *n*=12 per group and during resident-intruder: *t*_18_=2.155, *P*=0.045, *n*=10 per group). Error bars represent s.e.m. *P<0.05, **P<0.01, ***P<0.001 (Student’s *t*-test and one- or two-way analysis of variance followed by Bonferroni *post hoc* comparisons, where applicable). See also Supplementary Figs 1 and 2.

Rats were subjected to chronic restraint stress and, the next day, either prepared for nectin analyses or submitted to behavioural testing. To evaluate whether the effects depended on the chronic nature of stress, we also included a group just submitted to 1 day of the restraint stress paradigm. First, we verified that our western blots conditions to assess nectin-1 and nectin-3 levels were within the linear range (Supplementary Fig. 1). Then, we found that in the CA1 synaptoneurosomal fraction, there was a slight tendency for reduced nectin-1 expression (Fig. 1a,d) and a significant reduction of nectin-3 (Fig. 1b,d) following chronic, but not acute, stress. We also assessed the expression levels of N-cadherin, one known interacting partner of nectins, in the formation of cell–cell junctions^26^ and found reduced expression of N-cadherin in CA1 synaptoneurosomes again only following chronic stress (Fig. 1c,d). Importantly, no significant stress-induced changes were observed when nectin-1, nectin-3 or N-cadherin protein levels were measured in the whole homogenate (Supplementary Fig. 2a–d). In the CA3 region, no significant effect of stress was found on nectin-1 or nectin-3 protein expression in the synaptoneurosomal or whole homogenate fractions (Supplementary Fig. 2e–h). To further verify the specificity of the reduction in nectin-3 expression, we measured the expression levels of the cell adhesion molecule of the Ig superfamily, SynCAM-1, in the CA1 synaptoneurosomal fraction and found no significant changes following the stress regimes (Supplementary Fig. 2i).

At the behavioural level, sociability was reduced by chronic stress (Fig. 1e) but not following acute stress (Supplementary Fig. 2j). Social recognition tests rely on animals’ exploration of novel versus familiar conspecifics; however, locomotor activity in stressed subjects was indistinguishable from controls (Supplementary Fig. 2k) indicating that our findings are not confounded by a general behavioural inhibition. In the social memory test performed in the three-chambered apparatus, we found that although control animals preferentially explored the unfamiliar juvenile compared with the familiar animal, stressed animals did not differ in the exploration time devoted to the two juveniles (Fig. 1f). This social memory deficit was further verified in a free-moving paradigm with another cohort of chronically stressed rats (Fig. 1g). Finally, we confirmed previous observations^8^,^39^ indicating that chronic stress leads to increased aggressive behaviour, as indicated by the frequency of attacks against cagemates (Fig. 1h) and against unfamiliar males when tested in the resident-intruder test (Fig. 1i).

### Nectin-3 overexpression (OE) in CA1 prevents stress-induced alterations

**Figure 2:**
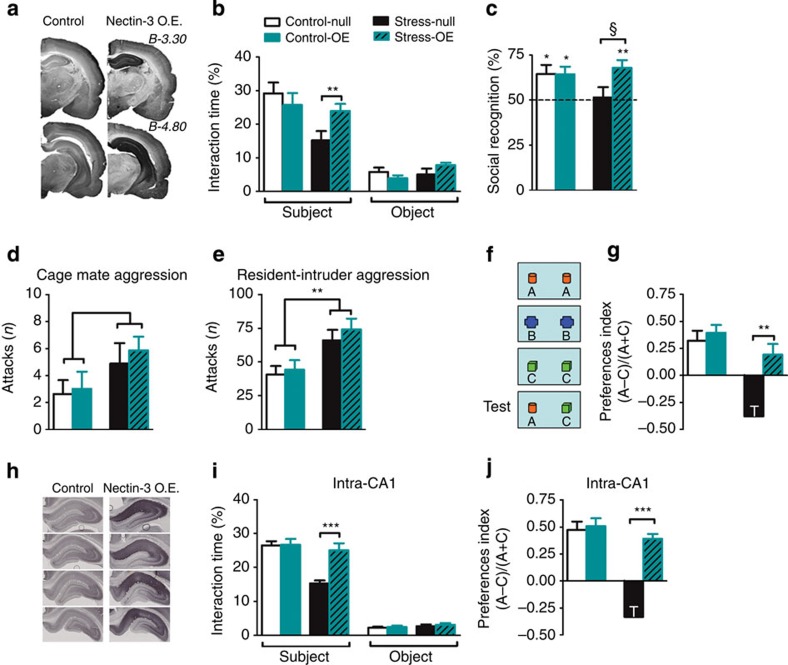
Nectin-3 overexpression (OE) prevents stress-induced impairments in sociability, social recognition and a CA1-dependent cognitive task. (**a**) Representative images showing the expression of nectin-3 5 weeks after intra-hippocampal injection of the empty AAV (null) or OE by nectin-3 AAV vectors (OE). (**b**) Stress reduces sociability but this is prevented by hippocampal nectin-3 OE (F_1,28_=7.187, *P*=0.0122, *n*=8 per group). (**c**) Social recognition was impaired in stressed animals and did not differ from chance level (50%) but hippocampal nectin-3 OE improved social recognition significantly (control-null: *t*_7_=2.873, *P*=0.0239, *n*=8; control-OE: *t*_6_=3.292, *P*=0.0166, *n*=7; stress-null: *t*_7_=0.2460, *P*=0.8127, *n*=8; stress-OE: *t*_7_=4.155, *P*=0.0043, *n*=8). (**d**,**e**) Aggressive behaviour against the cagemate and in the resident-intruder test was enhanced in stressed animals; hippocampal nectin-3 OE did not normalize this behaviour (cagemate: F_1,28_=4.357, *P*=0.0461, *n*=8 per group; resident-intruder: F_1,24_=13.4, *P*=0.0012, control-null *n*=6, control-OE *n*=7, stress-null *n*=8, stress-OE *n*=7). (**f**) Schema for the temporal order object discrimination task that specifically assesses intact CA1-functioning. (**g**) Stress affected the performance in this task but not after nectin-3 OE (F_1,28_=9.279, *P*=0.005, *n*=8 per group but *n*=6 for stress-null). (**h**) Representative images showing nectin-3 expression after intra-CA1 injection null or OE. (**i**,**j**) Intra-CA1 nectin-3 OE rescues impairments on sociability and performance on the CA1-dependent temporal order object discrimination task (sociability: F_1,52_=19.12, *P*<0.0001, control-null *n*=14, control-OE *n*=14, stress-null *n*=16, stress-OE *n*=12; object discrimination task: F_1,50_=37.7, *P*<0.0001, control-null *n*=12, control-OE *n*=14, stress-null *n*=16, stress-OE *n*=12). Error bars represent s.e.m. ^§,^*P<0.05, **P<0.01, ***P<0.001 (Student’s *t*-test, one-sample *t*-test and two-way analysis of variance followed by Bonferroni *post hoc* comparisons, where applicable). See also Supplementary Figs 3 and 4.

To determine whether reduced CA1 nectin-3 expression was causally related to the observed behavioural changes following chronic stress exposure, we employed an adeno-associated virus (AAV)-mediated gene delivery approach to induce site-specific nectin-3 OE. First, we targeted the whole hippocampus, as hippocampus-wide nectin-3 OE was recently shown to compensate for early life stress-induced deficits in hippocampus-dependent memory^25^. We confirmed by immunohistochemistry the validity of the AAV-nectin-3 serotype 1/2 to increase nectin-3 expression (Fig. 2a; Supplementary Fig. 3a). We could verify that the AAV-nectin-3 treatment did not affect the stress response, as indicated by similar plasma corticosterone levels in the AAV-empty (null) and nectin-3 OE-treated rats on day 10 of the chronic stress procedure (Supplementary Fig. 3b). In addition, the potential effects of the treatment on anxiety or exploration were discarded, as no significant differences were observed between the AAV control and AAV-nectin-3-treated rats in the open field (OF) and novel object (NO) reactivity tests (Supplementary Fig. 3c,d). We found that the body weight was only affected by the stress exposure and not by the AAV-nectin-3 treatment (Supplementary Fig. 3e). A bedding preference test discarded alterations in olfaction due in nectin-3 OE animals (Supplementary Fig. 3f).

At the behavioural level, stress again significantly reduced sociability, whereas nectin-3 OE did not have a main effect. Importantly, we observed a stress × AAV interaction (Fig. 2b), with nectin-3 OE reversing the stress effects in social exploration. Furthermore, nectin-3 OE rescued stress-induced impairments in social recognition for a familiar versus an unfamiliar juvenile (Fig. 2c). Thus, AAV-induced nectin-3 OE counteracted the alterations in social investigation and social memory induced by chronic stress. Nonetheless, nectin-3 OE did not reverse the potentiating effects of stress on aggression neither against homecage mates (Fig. 2d) nor in the resident-intruder test (Fig. 2e).

Given that the reduction in nectin-3 expression by chronic stress was specifically found in the CA1 area (Fig. 1b), we tested animals in the ‘temporal order memory for the visual object information task’ (termed hereafter ‘temporal order task’, see Fig. 2f), shown to depend on CA1 function^40^. This task is sensitive to CA1, but not to CA3, lesions; when given a preference for an object that was explored earlier or one that was explored later, controls chose the earlier, whereas CA1-lesioned rats chose the latter^40^. We found a significant virus × stress interaction (Fig. 2g). In agreement with our hypothesis, stress impaired performance in this task, whereas control animals (regardless of the viral treatment) preferred the early explored to the last explored object. Importantly, this effect was prevented by nectin-3 OE (Fig. 2g). At the end of the experiment, we performed immunohistochemical analyses to evaluate the content of nectin-1 and the synaptic marker, synaptophysin, in the hippocampus. Although nectin-3 OE increased nectin-1 expression in CA1 and CA3, irrespective of stress treatment (Supplementary Fig. 3g,h), synaptophysin levels were unchanged (Supplementary Fig. 3i,j).

Next, we investigated the specificity of nectin-3-related changes occurring in CA1 in stress-induced behavioural alterations. To this end, we set up the experimental conditions to confine nectin-3 OE to the CA1 region (Fig. 2h and Supplementary Fig. 4a). Again, nectin-3 OE did not affect corticosterone response or bodyweight changes induced by repeated stress exposure (Supplementary Fig. 4b,c). Remarkably, CA1-specific nectin-3 OE reproduced the effects on behaviour observed in the experiments described above in which we employed hippocampal-wide nectin-3 OE. Thus, overexpressing nectin-3 specifically in the CA1 area was sufficient to reverse the 21-day stress-induced deficits in social exploration (Fig. 2i) and in the temporal order task (Fig. 2j).

### Increased gelatinolytic activity in the CA1 of stressed animals

**Figure 3:**
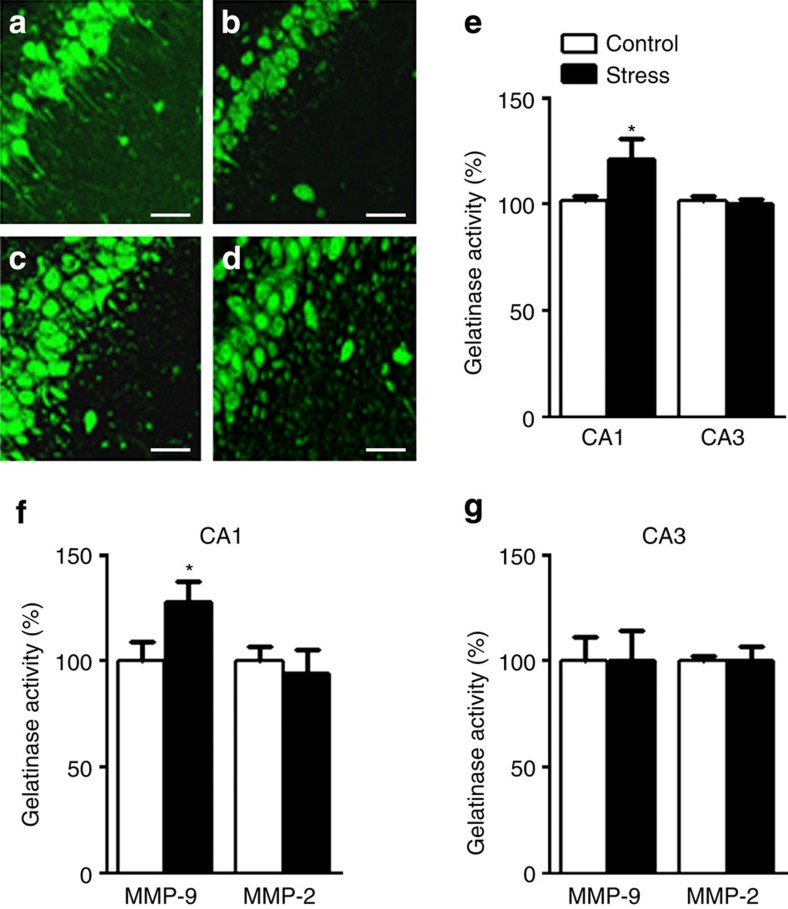
Chronic restraint stress enhances MMP-9-mediated gelatinase activity. (**a**–**d**) Representative confocal images of fluorescent *in situ* zymography from control and 21-day stressed animals in CA1 (**a**,**b**) and CA3 (**c**,**d**). (**e**) Gelatinase activity was increased in the CA1 but not in the CA3 (CA1: *t*_7_=2.63, *P*<0.05; CA3: *t*_14_=0.5530, *P*=0.5890, *n*=8 per group) and (**f**) was caused by enhanced MMP-9 activity, whereas MMP-2 activity was unaffected (MMP-9: F_22_=2.118, *P*=0.0457; MMP-2: F_22_=0.4496, *P*=0.6574, *n*=12 per group). (**g**) Stress did not affect MMP-9 or MMP-2 gelatinase activity in the CA3 (MMP-9: *t*_6_=0, *P*=1; MMP-2: *t*_6_=0, *P*=1, *n*=4 per group). Error bars represent s.e.m. *P<0.05 (Student’s *t*-test). Bar (**a**–**d**) represents a distance of 50 μm.

As we observed decreased expression of nectin-3 in synaptoneurosomal, but not in the total fraction, following chronic stress, we obtained support for the potential existence of membrane-shedding involvement in proteolytic activity. Thus, we determined the gelatinolytic *in situ* activity produced by MMP-2 and MMP-9 (ref. 13) in the hippocampus of rats submitted to chronic stress. Brain sections obtained from control and stressed animals were incubated with a fluorogenic substrate DQ gelatin (Fig. 3a–d). We found increased gelatinase activity in the cell bodies and neuropil in the CA1 region, but not in CA3 (Fig. 3e). Gel zymography experiments on the synaptoneurosomes proved that the CA1-related increased gelatinase activity stemmed from enhanced MMP-9 activation, whereas MMP-2 activity was not affected (Fig. 3f). As expected, both MMP-9 and MMP-2 activity in the CA3 were not altered by stress (Fig. 3g).

### NMDA-mediated cleavage of nectin-3 is dependent on MMP-9 activity

**Figure 4:**
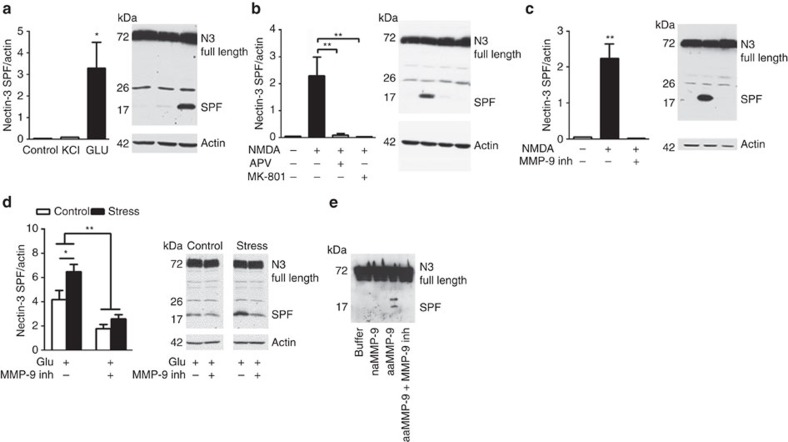
Nectin-3 cleavage is induced by glutamate and is NMDA- and MMP-9 dependent. Data in **a**–**c** are derived from three independent experiments. (**a**,**b**) Hippocampal neurons at day 7 were stimulated *in vitro* with KCl, glutamate or NMDA for 30 min in the presence or absence of inhibitors. Cleavage of full nectin-3 (83 kDa) results in the accumulation of a SPF (small proteolytic fragment). Nectin-3 cleavage was observed after glutamate and NMDA, whereas NMDA-induced nectin-3 shedding was inhibited in the presence of the NMDA-inhibitor APV (**a**: F_2,6_=6.25, *P*=0.04; **b**: F_2,14_=15.05, *P*<0.001). (**c**) Incubation of the hippocampal neurons with a MMP-9 inhibitor abolished the NMDA-induced nectin-3 cleavage (F_2,13_=13.76, *P*<0.001). (**d**) In synaptoneurosomes obtained from the CA1 from controls and stressed animals, we found that glutamate-induced nectin-3 shedding was more pronounced in stressed animals and that this effect could be prevented by application of a specific MMP-9 inhibitor (effect of stress: F_1,28_=33.71, *P*<0.0001; effect of MMP-9 inhibition: F_1,28_=7.797, *P*=0.0093, *n*=8 per group). (**e**) Cleavage of recombinant nectin-3 by MMP-9 *in vitro*. Recombinant nectin-3 was incubated with purified recombinant non-active MMP-9 (E402A), autoactivating MMP-9, autoactivating MMP-9 in the presence of 5 μM MMP-9 inhibitor, or no proteinase (buffer) as indicated. The digestion products were resolved by western blot analysis and probed with anti-His-Tag antibody. Error bars represent s.e.m. *P<0.05, **P<0.01 (one- or two-way analysis of variance followed by Bonferroni *post hoc* comparisons, where applicable).

Stress increases extracellular glutamate levels in the hippocampus^41^. To determine whether nectin-3 cleavage may result from enhanced neuronal activity, cultured hippocampal neurons were treated with either 30 mM KCl or 50 μM glutamate and extracts from whole-cell lysates were analysed via western immunoblotting. We found that glutamate treatment resulted in a significant increase in the level of the cleaved form of nectin-3 (small proteolytic fragment; Fig. 4a). This cleavage was dependent on NMDA receptor function, as it was reproduced by treatment with 100 μM NMDA and could be entirely inhibited by the NMDA receptor antagonists APV (DL-2-amino-5-phosphonopentaoic acid) or MK-801 (Fig. 4b).

To verify whether MMPs are responsible for the cleavage of nectin-3 after neuronal stimulation, neurons were pretreated with a specific MMP-9 inhibitor for 30 min before NMDA stimulation. NMDA-dependent stimulation of nectin-3 cleavage was abolished in the presence of the MMP-9 inhibitor (Fig. 4c).

To explore whether chronic stress exposure leads to a CA1-specific vulnerability for glutamate-induced nectin-3 cleavage mediated by MMPs, we performed experiments similar to the above in hippocampal subfield-specific synaptoneurosomes obtained from animals submitted to the chronic stress protocol (Fig. 4d). In CA1 synaptoneurosomes, the glutamate-induced increase in nectin-3 cleavage was enhanced in samples from stress as compared with controls (Fig. 4d). Glutamate-induced shedding of nectin-3 was inhibited in the presence of the MMP-9 inhibitor in both groups (Fig. 4d). Increased glutamate-evoked nectin-3 cleavage in synaptoneurosomes in the hippocampal CA1 of stressed animals was found under conditions that lead to increased gelatinase activity.

To confirm that MMP-9 is responsible for nectin-3 cleavage, recombinant nectin-3 was incubated either alone or with MMP-9 for 16 h. The partial cleavage of nectin-3 with MMP-9 resulted in a digestion product of approximately 20 kDa, corresponding to the molecular mass of small proteolytic fragment of nectin-3 (Fig. 4e). In contrast, proteolysis was observed neither under control conditions (buffer) nor in the presence of an inactive mutant of MMP-9, confirming the specificity of the assay. Moreover, shedding of nectin-3 was abolished by 5 μM MMP-9 inhibitor, confirming that MMP-9 was responsible for nectin-3 cleavage (Fig. 4e).

### MMP-9 inhibition prevents stress-induced behavioural alterations

**Figure 5:**
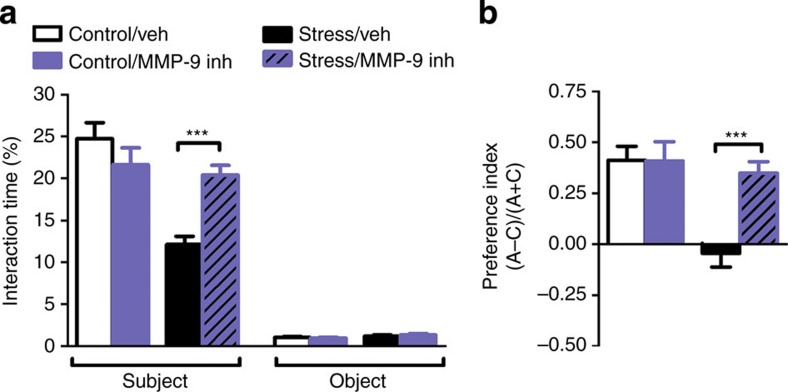
MMP-9 inhibition prevents stress-induced impairments in sociability and in temporal order object discrimination. (**a**,**b**) The intra-CA1 infusion of a specific MMP-9 inhibitor was sufficient to prevent stress-induced impairments in sociability and normalizes the performance in the CA1-dependent temporal order object discrimination task (sociability: F_1,57_=16.98, *P*=0.0001 control/vehicle (veh) *n*=10, control/MMP-9 inh *n*=14, stress/veh *n*=17, stress/MMP-9 inh *n*=18; object discrimination: F_1,44_=13, *P*=0.0008, control/veh *n*=12, control/MMP-9 inh *n*=10, stress/veh *n*=14, stress/MMP-9 inh *n*=12). Error bars represent s.e.m. ***P<0.001 (two-way analysis of variance followed by Bonferroni post hoc comparisons). See also Supplementary Fig. 5.

Our zymography and cell culture experiments pointed to MMP-9 as the mechanism mediating chronic stress-induced CA1 nectin-3 cleavage. To investigate this possibility, we performed intra-CA1 microinfusions of a novel specific MMP-9 inhibitor (MMP-9 PEX inhibitor) following a 2 × 2 experimental design. First, we verified the effectiveness of the MMP-9 inhibitor used to inhibit *in vivo*, the cleavage of β-dystroglycan, a well-known MMP-9 substrate^42^ (Supplementary Fig. 5a). Then, we investigated the relevance of MMP-9 activity in the CA1 in stress-induced behavioural effects by local infusion of a specific MMP-9 inhibitor. Although we show again that stress impairs sociability and CA1-related object recognition in the temporal order task, the infusion of a MMP-9 inhibitor prevented the emergence of these deficits (Fig. 5a,b) indicating a central role for MMP-9 in the pathophysiology of these stress-induced behavioural abnormalities. The treatment with the MMP-9 inhibitor did not affect the corticosterone response (Supplementary Fig. 5b) or body weight (Supplementary Fig. 5c) during chronic stress exposure, or olfaction as indicated by the results in the bedding preference test (Supplementary Fig. 5d).

### NMDA inhibition prevents the occurrence of stress effects

Our cell culture experiments also suggested that NMDA activation might underlie the stress effects induced by nectin-3 cleavage. To evaluate the implication of NMDA receptor in the stress-induced changes in nectin-3 expression and behavioural outcomes, we performed two studies in which we antagonized NMDA receptors *in vivo* during the chronic stress exposure utilizing a 2 × 2 experimental design involving stress and the NMDA inhibitor MK-801 as the factors. First, we gave the NMDA-inhibitor intraperitoneally (i.p.) and then performed a follow-up experiment in which it was microinfused into the CA1.

**Figure 6:**
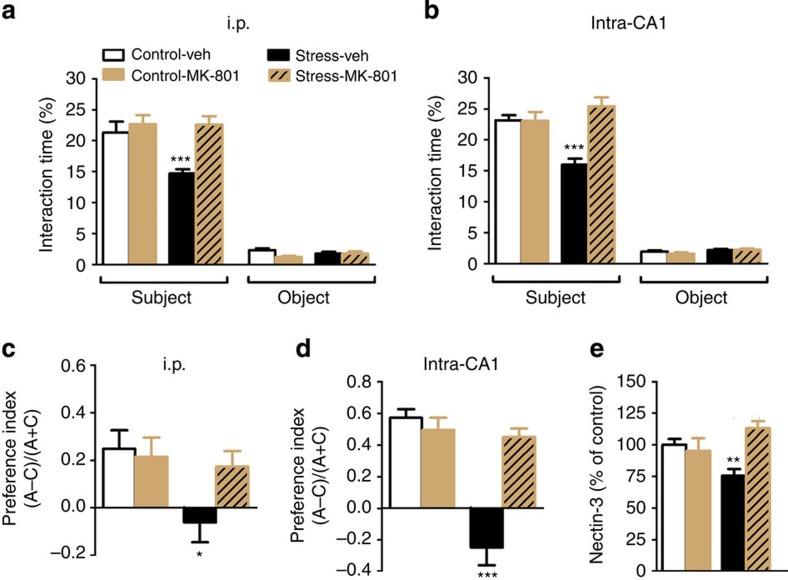
MK-801 prevents stress-induced impairments in sociability and in temporal order object discrimination. (**a**) Intraperitoneal (i.p.) injection of MK-801 normalizes the effects of chronic stress on sociability (F_1,35_=10.87, *P*=0.0022, *n*=10 per group but *n*=9 for stress-vehicle (veh)). (**b**) Although the performance in the CA-1-dependent temporal order object discrimination task is impaired by stress, MK-801 prevents this impairment (F_1,31_=12.56, *P*=0.0355, control-veh *n*=12, control-MK-801 *n*=12, stress-veh *n*=18, stress-MK-801 *n*=18). Intra-CA1 treatment of MK-801 recapitulated peripheral MK-801 treatment on both (**c**) sociability (F_1,56_=14.53, *P*=0.0005, control-veh *n*=12, control-MK-801 *n*=12, stress-veh *n*=18, stress-MK-801 *n*=18) and (**d**) temporal order object discrimination (F_1,49_=15.7, *P*<0.001, control-veh *n*=10, control-MK-801 *n*=10, stress-veh *n*=6, stress-MK-801 *n*=9). (**e**) MK-801 i.p. normalized nectin-3 expression in synaptosomes from the CA1 (interaction: F_1,27_=10.19, *P*=0.0036, control-veh *n*=10, control-MK-801 *n*=7, stress-veh *n*=6, stress-MK-801 *n*=8; i.p. treatment: *n*=6–10 per group, intra-CA1 treatment: *n*=12–18 per group). Error bars represent s.e.m. *P<0.05, **P<0.01, ***P<0.001 (two-way analysis of variance followed by Bonferroni *post hoc* comparisons, where applicable). See also Supplementary Fig. 6.

The reduction in sociability induced by chronic stress was abolished by MK-801 treatment both after i.p. (Fig. 6a) and after intra-CA1 infusion (Fig. 6b). The treatments did not influence locomotor activity in this test (Supplementary Fig. 6a). In the temporal order task, the reduced preference for the earlier versus the more recent object induced by stress was reversed by the MK-801 treatments (Fig. 6c,d). The protective effects of MK-801 treatments were not due to changes in the stress responsiveness (Supplementary Fig. 6b,c). MK801 treatment did not affect bodyweight (Supplementary Fig. 6d) or bedding preference (Supplementary Fig. 6e). Importantly, the reduction in CA1 synaptoneurosomal expression of nectin-3 following stress was reversed by MK-801 treatment (Fig. 6e).

## Discussion

We tested the hypothesis that MMP gelatinase activity is involved in key proteolytic processing events induced by chronic stress in a hippocampal subfield-dependent manner and in connection with behavioural changes. We show that chronic stress leads to a CA1-specific reduction in the perisynaptic expression of nectin-3 and found that this reduction is critically involved in the stress-induced deficits in social exploration, social recognition and CA1-dependent cognition. Interestingly, we found increased MMP-9-related gelatinase activity in the hippocampal CA1 in chronically stressed animals and could show that MMP-9 itself cleaves recombinant nectin-3, a process mediated via the NMDA-receptor. Consistently, intra-CA1 administration of either an MMP-9 inhibitor or an NMDA receptor antagonist during stress exposure prevented the development of stress-induced deficits in social exploration, social memory and CA1-dependent cognition. Our findings highlight a fundamental role for MMP-9 in the effects of chronic stress on brain function and behaviour.

Nectins are emerging as both targets^24^,^43^ and mediators^25^ of stress actions in hippocampal-dependent memory and structural plasticity. We found molecular-, regional-, cellular compartment- and stress duration-dependent changes, with reduced nectin-3 expression after 21 days, but not 1 day, of restraint stress in the CA1 synaptoneurosomal, but not the total fraction. This was paralleled by deficits in several social behaviours and in a CA1-dependent cognitive task. Our results from cell culture experiments suggested that NMDA receptor activation during stress exposure might be implicated in the cleavage of nectin-3 in CA1 and its associated behavioural alterations. Previous work has implicated NMDA receptor activation in chronic stress-induced structural alterations in the hippocampus^12^,^44^,^45^. Our *in vivo* study involving the pharmacological administration of the NMDA receptor antagonist MK-801, either systemically or directly into the CA1 region, confirmed that this treatment prevented the stress-induced reduction of nectin-3 expression in the CA1 synaptoneurosomal fraction as well as the behavioural impairments induced by stress in the sociability and temporal order task.

Using AAV-induced OE of nectin-3 either in the whole hippocampus or specifically in the CA1 area, we obtained evidence for a causal role of nectin-3 reduction in chronic stress-induced behavioural alterations, with the exception of the aggressive phenotype. We confirmed that the effects of nectin-3 OE were not due to altered physiological responses to the stress procedure (for example, body weight changes or corticosterone responses) or to changes in anxiety or locomotion. We found increased nectin-1 expression associated with AAV-nectin-3 OE throughout the hippocampus, consistent with evidence in knockout mice indicating that downregulation of either nectin-1 or nectin-3 induces a parallel decrease in the levels of the other nectins in the hippocampus^46^. Synaptophysin levels were not changed by nectin-3 OE and/or chronic stress, which is line with findings described for nectin-3 knockout mice^46^. In addition, using the same chronic restraint stress protocol as described here, changes in the size of postsynaptic densities were observed but not in synaptic density in the CA1 (ref. 5). Interestingly, consistent with evidence that nectins recruit cadherins to cooperatively promote cell adhesion^47^, we found a reduction in the CA1 perisynaptic N-cadherin levels. The specificity of these molecular changes in CA1 was supported by a lack of significant changes in the stressed animals’ synaptoneurosomal compartment of SynCAM-1 in the same brain region. To verify that the molecular changes specifically observed in CA1 were associated with well-established CA1-dependent behaviours, we tested animals in the temporal order task that is sensitive to CA1, but not to CA3, lesions^40^. With regard to region-specificity, our findings for CA1 are in contrast with recent evidence in mice showing reduced nectin-3 expression in CA3 (refs 24, 25). This disparity may be attributed to differences in the animal species or stress procedures.

MMPs are a family of proteolytic enzymes that degrade components of the extracellular matrix and cleave specific cell-surface proteins^48^, making them particularly suitable to sustain neural remodelling processes^15^. The degradation of cell adhesion molecules is one of the main mechanisms whereby MMPs affect neural plasticity^9^,^22^ and the synapse-associated nectin-3 decrease suggested the potential involvement of proteolytic processing. Nectin-1 has been shown to undergo ectodomain shedding by alpha-secretase^32^; however, the molecular players involved in nectin-3 shedding remained unknown.

We found that decreased nectin-3 expression in the hippocampal CA1, but not in the CA3, synaptoneurosomes of the stressed animals is accompanied by increased gelatinase activity. This suggested an increase in MMP-2 and/or MMP-9 activity, as these two MMPs are the most prominent gelatinases expressed in the brain. Our cell culture experiments also indicated that NMDA receptor stimulation leads to increased nectin-3 proteolytic cleavage that is MMP-9 dependent. The involvement of MMP-9 and not MMP-2 is consistent with a previous study showing that MMP-2 does not interact with nectin-3 (ref. 49). Furthermore, we provide direct evidence that MMP-9 cleaves recombinant nectin-3. Interestingly, MMP-9 cleaves several postsynaptic proteins involved in trans-synaptic adhesion via their interaction with presynaptic proteins. The list of such MMP-9 targets includes β-dystroglycan that binds to neurexins^42^ as well as neuroligin-1 also binding neurexins^50^. Our findings are in line with previous reports implicating hippocampal MMP-9 in changes in dendritic spine morphology^51^ as well as in the cellular processes that contribute to a stressful learning task^20^. Importantly, we show that intra-CA1 treatment with a specific MMP-9 inhibitor prevented the emergence of chronic stress-induced effects in social exploration and CA1-dependent cognition. Therefore, our results are consistent other findings that indicate a crucial role for extracellular proteolysis in the stress-induced behavioural alterations, with former studies highlighting the role of serine proteases, including the tissue-plasminogen activator^12^ and neuropsin^10^.

Although deregulated social behaviour is a hallmark of many psychiatric disorders^52^, studies focusing on the link between chronic stress and psychopathology has mainly concentrated on studies in mood and cognition^2^,^7^, whereas the effects of stress on social behaviours are much less known. In agreement with our previous study^8^, we confirm here that chronic restraint stress for 21 days leads to clear alterations in the social domain, including reduced sociability, impaired social memory and increased aggressive behaviours. The hippocampus has been implicated in social behaviours both in rodents^53^ and in humans^54^. Consistent with our findings, social recognition in rats was disrupted by CA1 damage^55^. However, although the effects of stress on sociability and social memory were rescued with nectin-3 OE, increased aggressive behaviours were not modified by this treatment. We have recently found that targeting the cell adhesion molecule neuroligin-2 expression or function in the hippocampus alters aggressive behaviour^8^,^56^, suggesting the involvement of the hippocampus in the regulation of aggression. However, it should be noted that those treatments were not confined to the CA1 area, which, on its own, might not modulate aggressive behaviours.

In summary, our findings identify a key role for MMP-9 proteolytic processing of nectin-3 in the hippocampal CA1, through a mechanism that engages NMDA receptors, among the processes leading to chronic stress-induced changes in social and cognitive behaviours. In addition to nectin-3, recently identified as potential mediators in stress-related disorders^25^, our study highlights MMP-9 activity as a novel target for the treatment of stress-related neuropsychiatric disorders, in particular depression, which is typically characterized by deficits in the social and cognitive domains.

## Methods

### Animals

Adult male Sprague–Dawley rats (Charles River Laboratories) were pair-housed with *ad libitum* access to food and water under a light–dark cycle (12:12 h, lights on 0800–2000 hours) and temperature (21±1 °C)-controlled conditions. Experimenters were blinded to the treatment groups, sample sizes were based on previous experiments and/or a power analyses. All procedures were conducted in accordance with the Swiss National Institutional Guidelines of Animal Experimentation and were approved by the Swiss Cantonal Veterinary Office Committee for Animal Experimentation (license numbers 2385 and 2145).

### General behavioural procedures

Both cagemates received the same experimental treatments throughout the study, and their body weight was measured periodically. As trait anxiety is identified as a vulnerability factor to develop depression when facing stress^57^, we matched animals for anxiety based on behaviour in the elevated plus maze, the OF and NO reactivity tests before the start of the experiments (data not shown). In the experiments that involved AAV-induced nectin-3 OE, the groups were created on the basis of the elevated plus maze results that were obtained before the virus infusion, and the OF and NO tests were performed post-surgery to verify the absence of the effects of nectin-3 OE on the rats’ emotional and exploratory behaviours. After each test, the apparatuses were cleaned with 1% acetic acid solution. The Noldus Ethovision software (Noldus Information Technology) was used to analyse locomotor-related behaviours and time spent in zones. Other behavioural interactions were analysed from video-recorded events using the Observer program (version 5.0, Noldus Information Technology).

### Restraint stress procedure

Rats were gently wrapped in a paper towel to minimize the risk of injuries and restrained 6 h per day for either 1 or 21 days in wire mesh restrainers (26 × 26 cm^2^) in their home cages. There was no indication of wounds or injuries to the rats.

### The social approach and social memory tasks

The sociability test was performed in a rectangular, three-chambered box that included a central compartment where the rat was initially placed and the left and right compartments where the choices were presented. Next, two memory tests were given. The first one was performed in the same three-chambered apparatus, 30 min after the social preference test. The unfamiliar object was replaced with a novel juvenile. The time the rat spent sniffing the juvenile that it had previously encountered during the social preference test (familiar) or the novel one (unfamiliar) was scored. The second memory test was adapted from a procedure as described^58^. Animals were tested in their home cage during 5-min exposure to a juvenile. This was followed by a second exposure to the same or a different juvenile 30 min after the first exposure.

### Social memory test

Briefly, a social discrimination session consisted of three 4-min exposures of juveniles to the adult in a clean cage with fresh bedding. During the first exposure (‘sampling’), the juvenile was exposed to the adult animal and was then removed and kept individually in a fresh cage with food and water available *ad libitum*. After a defined retention interval of 30 min, the same juvenile (now familiar) was re-exposed to the experimental adult for 4-min period (second exposure). To assess whether the reduced investigation duration during the second exposure reflects the juvenile-related memory, an additional 4-min session was performed 30 min later in which the experimental rat was exposed to a novel juvenile (third exposure). Each trial was recorded using a video camera that was equipped with an infrared light source for low-light conditions. Furthermore, the duration of the investigative behaviour of the adult towards the juvenile (mainly sniffing and licking of the anogenital region of the juvenile) was recorded.

### Temporal order for the visual object task

The rats were placed inside a grey plastic box (40 cm across × 60 cm length × 32 cm depth) to explore each set of three objects (referred to as A-A, B-B and C-C) for 5 min, with a 3-min inter-session interval. The objects were placed so that the distance was far apart and there would be no ambiguity as to which object the rat was exploring. After the third set of objects, the rats were given a 3-min time-out, after which a third copy of object A and a third copy of object C were placed in the opposite ends of the box. The rats were then returned to the box to measure their preference for object A versus object C for a 5-min period. To determine the overall activity level of each rat for the temporal order task, the preference ratio of the difference between object A and object C divided by the sum of object A and object C was calculated. A zero score would reflect no preference for objects A or C.

### Social aggression tests

The resident-intruder test and the social aggression in a neutral cage were performed as previously described^8^. In the resident-intruder test, male rats were housed together with a female rat for 10 days in an experimental cage. The female was removed 30 min before the test. The test was then performed during the beginning of the dark cycle (between 2000 and 2200 hours). The resident experimental male was exposed in its home-cage with a slightly smaller (5% lighter) unfamiliar male Sprague–Dawley rat for 30 min. Several parameters related to male aggression were scored and the total number of attacks computed. Social aggression was scored based on 15-min videos starting 5 min after the animals’ release from restrainers on day 21, the last day of the stress procedure. For the control rats, a video was taken at the same time of the day after a brief handling. Several parameters related to male aggression were scored and the total number of attacks was computed.

### Bedding preference test

This test was described previously^59^. Briefly, each experimental animal was placed in the centre of an OF arena (see description above) where two Plexiglas cylinders (15 cm Ø, pierced with 5-mm Ø holes) were placed on opposite ends of the arena, in close proximity to the walls. One cylinder was filled with clean bedding, and the second was filled with mixed soiled bedding. The mixture of soiled bedding was collected from cages that contained two or three adult Sprague–Dawley males. There was no bedding on the floor of the testing apparatus. The animals were scored for time spent investigating (sniffing) each bedding stimulus during the 10-min test.

### Intracerebral cannulation

Rats were anaesthetized by isoflurane inhalation (induction: 4% isoflurane for 4 min, maintenance: 2.5% isoflurane in O_2_ at a flow of 4 l min^−1^) and placed in a stereotaxic apparatus (David Kopf Instruments). Small holes were drilled through the skull for bilateral placement of stainless steel 22-G guide cannulae (Plastics One) fitted with a removable dummy cannula. Coordinates aimed at the hippocampal CA1 were based on the atlas of Paxinos and Watson^60^ and are taken from bregma (in mm; AP=−3.60, ML=±2.8, DV=−1.93 at an 11° angle to avoid damaging the superior sagittal sinus). Cannulae were fixed to the skull with two anchoring screws and dental acrylic cement (Duralay 2244; Reliance). After behavioural experiments, animals were killed by i.p. pentobarbital injection and correct cannulae placements were routinely verified with Evans blue histology.

### Quantification of plasma corticosterone levels

On the day 10 of the chronic restraint stress, blood samples were obtained by the tail-nick method^61^ from the stressed animals under basal conditions and during the restraint stress period. Animals from different groups were counterbalanced along the sampling period. Blood plasma was stored at −20 °C until the corticosterone levels were assayed by ELISA (Assay Design). The inter-assay coefficient of variation was 8%.

### Drug administration *in vivo* experiments

Animals were treated on days 1, 2, 6, 9, 10, 13, 15, 20 and 21 of restraint stress. The non-competitive NMDA receptor antagonist MK-801 maleate (Biotrend) was injected i.p. (0.1 mg kg^−1^) or intra-CA1 (bilateral 6.25 μg/0.5 μl) 30 min before the onset of restraint stress and control rats were injected with an equal volume (1 ml kg^−1^ i.p. or 0.5 μl per side) of saline vehicle. The MMP-9 inhibitor (inhibitor II, Calbiochem) was dissolved in dimethylsulphoxide and infused intra-CA1 (bilateral 1 μg/0.5 μl).

### Drug administration *in vitro* experiments

Primary hippocampal cultures at 7 days* in vitro* (DIV) were stimulated with KCl (30 mM; Sigma), NMDA (100 μM; Sigma) or glutamate (50 μM; Sigma) in the presence or absence of inhibitors (APV – 100 μM; MK-801–50 μM Sigma and MMP-9 inhibitor I, 5 μM; Merck). All inhibitors were added 30 min before stimulation in co-treatment experiments.

### Cell cultures

Hippocampal neurons were prepared from newborn Wistar rats at postnatal day P0 as described previously with modifications^62^. Briefly, the culture medium consisted of Neurobasal supplemented with B27 (Invitrogen) and 1 mM L-glutamine, 100 U ml^−1^ penicillin and 0.1 mg ml^−1^ streptomycin. The cells used for these experiments were 7 DIV.

### Production and infusion of AAV vector

Hybrid AAV vectors derived from serotypes 1 and 2 encoding nectin-3 were prepared by GeneDetect. The viral cassette contained a hybrid cytomegalovirus enhancer/chicken-actin promoter (CBA), a woodchuck posttranscriptional regulatory element, a scaffold-attachment region and a bovine growth hormone polyadenylation sequence (bGH-pA). The mouse nectin-3 alpha expression plasmid was kindly provided by Yoshimi Takai (Osaka University, Osaka, Japan). The same backbone lacking a cDNA was used as a control (AAV-empty). The viral vectors were packaged and affinity purified (GeneDetect). The titre of the virus particles (=stock) was determined by reverse transcription–PCR at a concentration of 5.2 × 10^11^ genomic copies per ml.

The AAV vectors were injected under ketamine/xylazine anaesthesia in rats weighing approximately 300 g using a 34-G needle in two bilateral spots aimed at the dorsal hippocampus (anterior-posterior (AP)=−3.5, medial-lateral (ML)=±1.5 from Bregma and dorsal-ventral (DV)=−3.5 from skull surface). 2 μl of stock solution was bilaterally infused at 0.2 μl min^−1^ using a 10-μl Hamilton syringe (Hamilton Bonanduz AG), mounted to an automatic pump (CMA Microdialysis). For the targeting of the dorsal hippocampal CA1 (AP=−3.5, ML=±1.5 and ±2.5 from Bregma and DV=−3.1 from skull surface), 0.7 μl of stock solution was bilaterally infused at 0.2 μl min^−1^. The needle was left in place for an additional 5 min before being slowly withdrawn.

### Preparation of total fraction and synaptoneurosome samples

The synaptoneurosomal and total fraction were prepared as described^63^. Tissue samples were homogenized in ice-cold homogenization buffer (10 mm HEPES, 1.0 mM EDTA, 2.0 mM EGTA, 0.5 mM dithiothreitol, 0.1 mM phenylmethanesulfonyl fluoride) containing a freshly added protease and phosphatase inhibitor cocktail (Complete EDTA free, Roche Diagnostics GmbH), with an Eppendorf homogenizer. At this stage, the aliquots of whole homogenates (total fraction) were solubilized with 1% NP-40, and the sample was centrifuged at 1,000 × *g* for 10 min. The debris was removed and stored at −80 °C for future analysis. The remaining homogenates were passed through two 100 μm-pore nylon mesh filters, followed by two 5-μm-pore filters. The filtered homogenates were then centrifuged at 1,000 × *g* for 10 min at 4 °C. The resultant pellets were resuspended in 100 μl 1% SDS, boiled for 1 min and stored at −80 °C. The whole and synaptoneurosome hippocampal samples were quantified using the detergent-compatible protein assay (Bio-Rad).

Equal protein samples were prepared at a concentration of 0.75 μg ml^−1^ in 33 mM NaCl, 70 mM Tris–HCl, 1 mM EDTA, 2% (w/v) SDS, 0.01% (w/v) bromophenol blue and 10% glycerol, pH 6.8. The proteins were resolved on 10% polyacrylamide gels and transferred to nitrocellulose membranes. The membranes were blocked for 1 h at room temperature with 5% non-fat dry milk in Tris-buffered saline (TBS)-0.1% Tween-20. The membranes were then incubated with primary antibody (Nectin-1, 1:5,000, SCBT, H-62 sc-28639; Nectin-3, 1:3,000, Abcam, ab63931; pan-actin 1: 20,000, Sigma and GAPDH, 1:100,000, Abcam, 6C5 ab8245) overnight at 4 °C. The membranes were subsequently washed three times in TBS-0.1% Tween-20 for 10 min and then incubated for 2 h at room temperature with the appropriate secondary horseradish peroxidase-linked antibodies diluted in blocking buffer. Following membrane washing with TBS-0.1% Tween-20 buffer, the immunocomplexes were visualized using a chemiluminescence peroxidase substrate (SuperSignal West Dura Extended Duration Substrate), and the immunoreactivity was detected using the ChemiDoc XRS system (Bio-Rad). A densitometry analysis of the bands was calculated using the Quantity One 4.2.3 software (Bio-Rad Laboratories AG). Each band was normalized to the GAPDH level as determined in the corresponding sample. On each gel, at least two naive controls were included. Protein changes are represented as the percentage of the normalized naive value. All immunoblot assays were performed within the linear range (see Supplementary Fig. 1).

### Stimulation of synaptoneurosomes

Synaptoneurosomes were stimulated with glutamate (50 μM, 30 min.) in the presence or absence of a selective MMP-9 inhibitor (inh.I, 5 μM). The inhibitor was added 30 min before stimulation in co-treatment experiments. After stimulation, synaptoneurosomes were lysed in the SDS sample buffer, denatured and fractionated on 12% SDS–polyacrylamide gels.

### Western blot analyses

In the experiments that involved western blot analyses, the rats were killed by decapitation and the CA1, CA3 and dentate gyrus of the hippocampal formation were quickly and manually dissected into an ice-cold Petri dish covered with a cold PBS-moistened filter paper, frozen in liquid nitrogen and stored at −80 °C. Synaptoneurosomes and total fraction were prepared as described previously^61^. The protein samples were prepared at a concentration of 0.75 μg ml^−1^ in 33 mM NaCl, 70 mM Tris–HCl, 1 mM EDTA, 2% (w/v) SDS, 0.01% (w/v) bromophenol blue and 10% glycerol, pH 6.8. The proteins were resolved on 10% polyacrylamide gels and transferred onto nitrocellulose membranes. The membranes were blocked for 1 h at room temperature with 5% non-fat dry milk in TBS/0.1% Tween-20. The membranes were then incubated with primary antibody (Nectin-1, 1:5,000, SCBT, H-62 sc-28639; Nectin-3, 1:3,000, Abcam, ab63931; pan-actin 1:20,000, Sigma and GAPDH, 1:100.000, Abcam, 6C5 ab8245) overnight at 4 °C. The membranes were washed three times in TBS-0.1% Tween-20 for 10 min and then incubated for 2 h at room temperature with the appropriate secondary horseradish peroxidase-linked antibodies diluted in blocking buffer. Following the membrane washing with TBS-0.1% Tween-20 buffer, the immunocomplexes were visualized using a chemiluminescence peroxidase substrate (SuperSignal West Dura Extended Duration Substrate), and the immunoreactivity was detected using the ChemiDoc XRS system (Bio-Rad). A densitometry analysis on the bands was calculated using Quantity One 4.2.3 software (Bio-Rad Laboratories AG), where each band was normalized to the GAPDH level determined in the corresponding sample. On each gel, at least two naive controls were used, and the protein changes were represented as a percentage of the normalized naive value (see Supplementary Fig. 1). The non-cropped blots for the representative images as shown in the main- and supplementary figures are displayed in Supplementary Fig. 7.

### Immunohistochemistry

Rats were anaesthetized with a lethal dose of pentobarbital and perfused transcardially using 0.9% saline solution followed by a fixative solution of paraformaldehyde 4% in PBS (pH=7.5). The brains were removed, post-fixed for 4 h in 4% paraformaldehyde/PBS and cryoprotected in 30% sucrose/PBS. Coronal sections (30 μm thick) were cut on a cryostat (CM3050 S), and an alternate series of one in ten free-floating sections were used for immunohistochemical visualization of nectin-1 and synaptophysin. The optical density was determined in every tenth section covering the entire hippocampus (an average of 12 sections).

The floating sections were rinsed briefly with Tris-buffered saline/0.1% Triton-X-100 (TBS-T), and endogenous peroxidases were blocked by incubation in 0.3% H_2_O_2_/TBS-T. After the TBS-T washes, the sections were blocked in 5% normal donkey serum/TBS-T and incubated overnight at 4 °C with antibodies against nectin-1 (1:1,000), nectin-3 (1:1,000) or synaptophysin (1:1,000). The sections were washed in TBS-T and incubated for 1 h at room temperature with biotinylated goat anti-rabbit IgG or goat anti-mouse IgG (both 1:200; Vector Laboratories). Images were taken with a digital camera that was coupled to a bright-field microscope (Olympus BX51) using a × 20 objective. The sample images were captured at the same coordinates for each animal. Seven to ten sample images of both hemispheres for each rat were used for the analysis. A densitometric analysis of the protein immunoreactivity was conducted using image analySIS pro 5.0 software (Olympus Soft Imaging Solutions GmbH). Briefly, the areas of interest were delimitated, and the optical densities in the different regions of interest were obtained. The background was subtracted from the density value of the sample taken in the corpus callosum of the same section, and analyses were performed blind to the experimental conditions.

### Nectin-3 *in vitro* cleavage assay

Recombinant nectin-3 protein (R&D Systems; #3064-N3; 40 ng) was incubated alone or with non-active MMP-9 (E402A; 100 ng), recombinant autoactivating MMP-9 (100 ng) or recombinant autoactivating MMP-9 in the presence of 5 μM MMP-9 inhibitor (Merck; #444252) in the assay buffer (50 mM Tris–HCl, pH 7.5, 150 mM NaCl, 10 mM CaCl2 and 0.5 mg ml^−1^ BSA) for 16 h at 37 °C. Reactions were terminated by adding SDS–polyacrylamide gel electrophoresis loading buffer and boiling for 5 min. Assay samples were subjected to 12% polyacrylamide gel electrophoresis, transferred to polyvinylidene difluoride membranes and immunoblotted with anti-His-Tag antibody (Abcam;# ab9108, 1:1,000).

### Gel zymography

Synaptoneurosomal fractionation for gel zymography was performed as described^17^. The synaptoneurosomes were subjected to electrophoresis in SDS–polyacrylamide gel electrophoresis Tris-glycine 8% acrylamide gels containing 0.5% gelatin (POCH) under nondenaturating, nonreducing conditions. Half of the samples were denaturated in 80 °C for 10 min before loading onto gel. Gels were washed twice for 30 min in 2.5% Triton X-100 and incubated in the zymography buffer (50 mM Tris, pH 7.5, 10 mM CaCl_2_, 1 μM ZnCl_2_, 1% Triton X-100 and 0.02% sodium azide) for 7 days at 37 °C. Gels were then stained with 0.5% Coomassie blue R-250 and de-stained with 5% acetic acid.

### Statistics

Data normality was checked using Shapiro–Wilk normality test and the D’Agostino-Pearson omnibus test. When normality was confirmed, data were analysed with either Student’s *t*-tests, one-sample *t*-tests against chance (50%) or analysis of variance (one- or two-way) followed by Bonferroni post-test, when appropriate. When normality was rejected, data were analysed with the Mann–Whitney test followed by Dunn’s multiple comparisons test. Significance was set at *P*<0.05.

## Author contributions

C.S., L.K., M.F. and M.A.v.d.K. designed and planned the experiments, which were conducted by J.G., O.Z. E.R, M.F., M.A.v.d.K., C.F., K.G. and K.K. Data analysis and drafting of the results were done by M.A.v.d.K., M.F., J.G., O.Z. and E.R. The paper was written by C.S., L.K., M.A.v.d.K and M.F. M.A.v.d.K., M.F., E.R., J.G. and O.Z. contributed equally to this study.

## Additional information

**How to cite this article:** van der Kooij, M. A. *et al.* Role for MMP-9 in stress-induced downregulation of nectin-3 in hippocampal CA1 and associated behavioural alterations. *Nat. Commun.* 5:4995 doi: 10.1038/ncomms5995 (2014).

## Supplementary Material

Supplementary Figures and Methods Supplementary Figures 1-7 and Supplementary Methods
