# Supplementary material for: Role for MMP-9 in stress-induced downregulation of nectin-3 in hippocampal CA1 and associated behavioural alterations
Source: Nat Commun. 2014 Sep 18;5:4995. doi: 10.1038/ncomms5995 (PMC4199199; doi:10.1038/ncomms5995)
Supplement: Supplementary Figures and Methods — Supplementary Figures 1-7 and Supplementary Methods [file ncomms5995-s1.pdf]

Supplementary information

Supplementary Figures

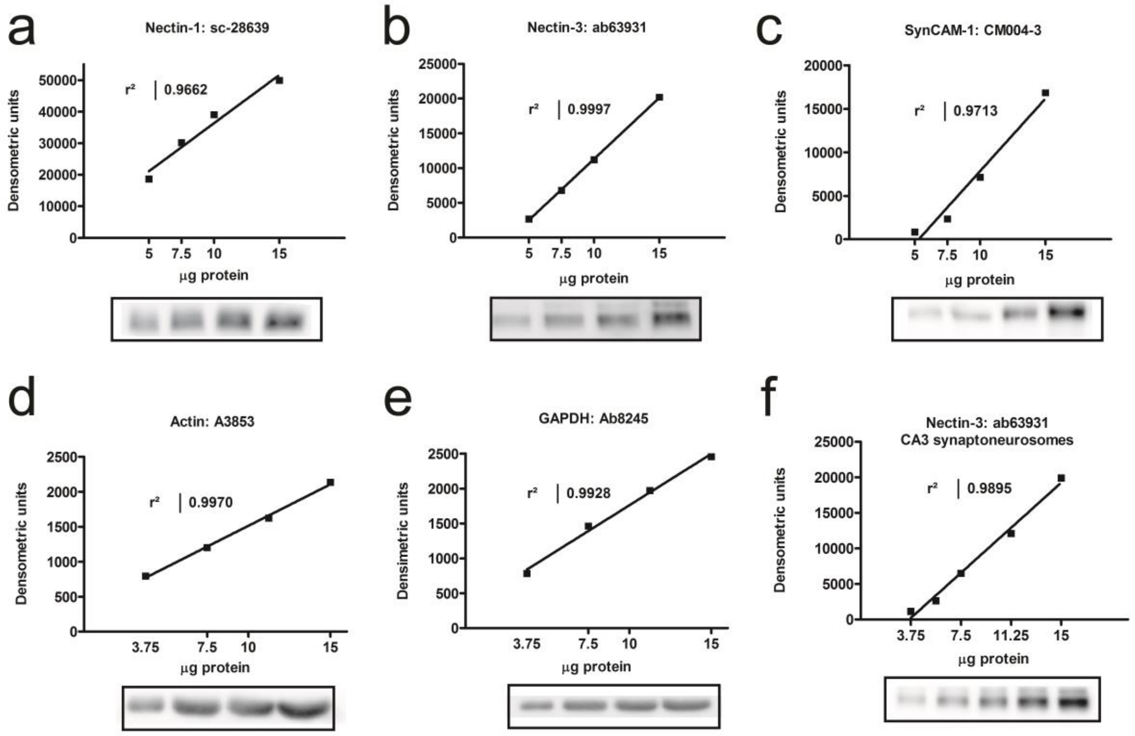

Supplementary Figure 1 The conditions to detect each synaptic protein (nectin-1, nectin-3, SynCAM-1, actin and GAPDH) within the linear range of the detection system were determined prior to application to quantify the protein content in the experimental samples. Linear correlations were found between the amount of protein loaded (concentrations ranging 3.75-15 µg) and the obtained signal intensity in the western blot. Representative immunoblots are displayed, and the graphs depict the chemiluminescent signal intensity plotted against the protein concentration for (**a**) nectin-1, (**b**) nectin-3, (**c**) SynCAM-1, (**d**) actin, (**e**) GAPDH and (**f**) nectin-3 in the CA3 synaptoneurosome preparation.

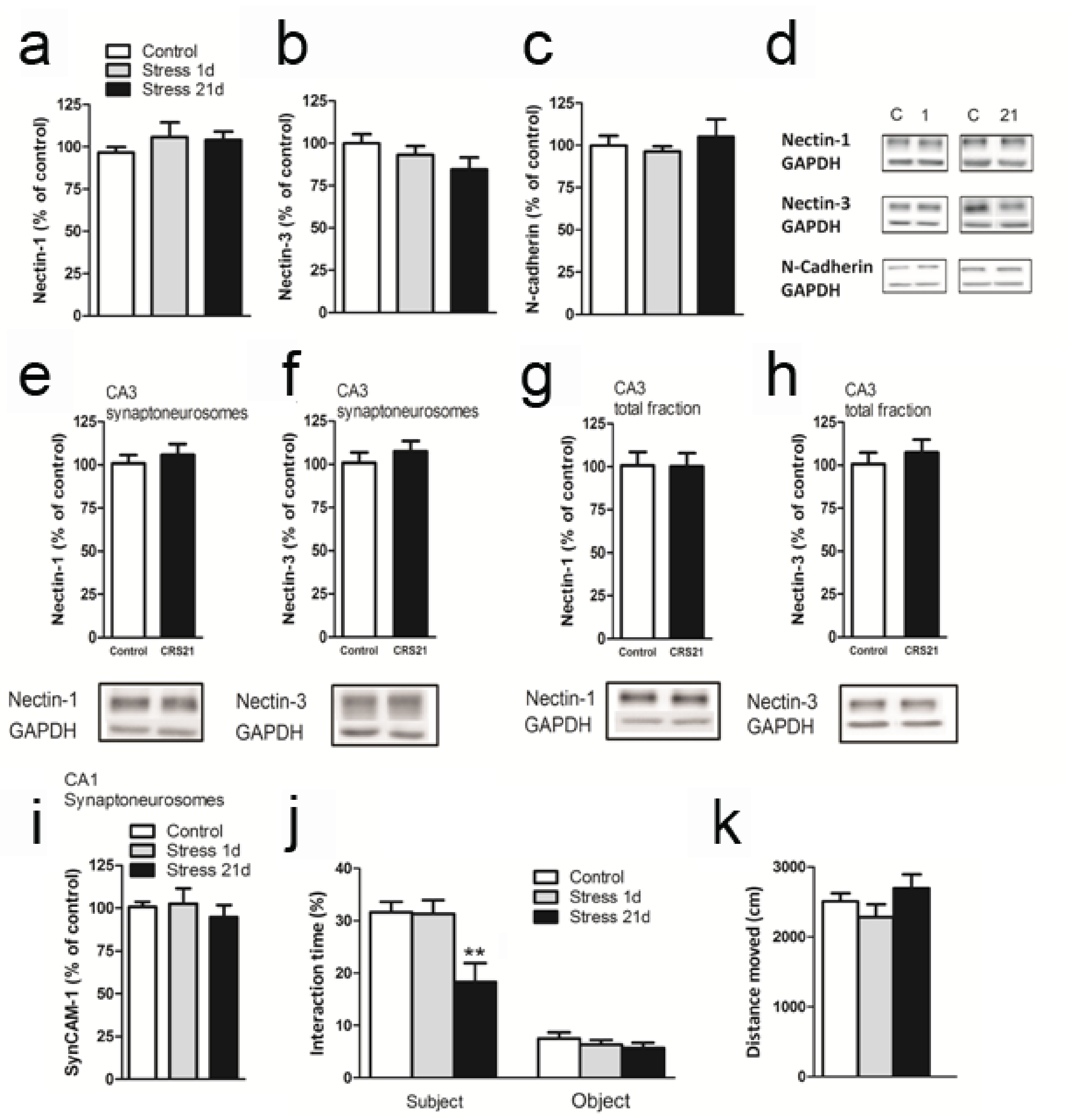

Supplementary Figure 2 (**a-c**) Nectin-1, Nectin-3 and N-cadherin protein expression in whole homogenates of the CA1 was not affected after 1 or 21 days of repeated restraint (nectin-1: $F_{2,49}$= 1.126, p= 0.3328, control n= 26, stress 1d n= 6, stress 21d n= 18; nectin-3: $F_{2,25}$= 1.719, p= 0.2015, control n= 12, stress 1d n= 4, stress 21d n= 10; n-cadherin: $F_{2,36}$= 0.209, p= 0.8126, control n= 20, stress 1d n= 5, stress 21d n= 12). (**d**) Representative western blots from CA1 homogenates. (**e**) nectin-1 and (**f**) nectin-3 protein levels were not modulated by stress in the synaptoneurosomes prepared from CA3 (nectin-1: t_{20}= 0.5508, p= 0.5508, control n= 10, stress 21d n= 12; nectin-3: t_{26}= 0.7768, p= 0.4443, control n= 16, stress 21d n= 12). (**g,h**) The total CA3 nectin expression levels were unchanged by stress in the total fraction of CA3 tissue (nectin-1: t_{22}= 0.03825, n= 12/group; nectin-3: t_{21}= 0.6755, p= 0.5068, control n= 11, stress 21d n= 12). (**i**) SynCAM-1 protein expression in CA1 synaptoneurosomal fraction was not affected by 1 or 21d of restraint stress ($F_{2,29}$= 0.469, p= 0.6305, control n= 14, stress 1d n= 6, stress 21d n= 10). (**j**) Social preference in the three-chambered apparatus was only affected for rats subjected to the 21d stress paradigm ($F_{2,24}$= 7.23, p= 0.0041, , control n= 11, stress 1d n= 8, stress 21d n= 6). (**k**) The distance moved in the three-chambered apparatus during the social preference test indicated no differences in the motor activity across the

different experimental groups ($F_{2,33}$= 1.18, p= 0.3206, control n= 20, stress 1d n= 8, stress 21d n= 6). Error bars represent SEM. **p<0.01 (Student's t-test, one- or two-way ANOVA followed by Bonferroni post hoc comparisons, where applicable).

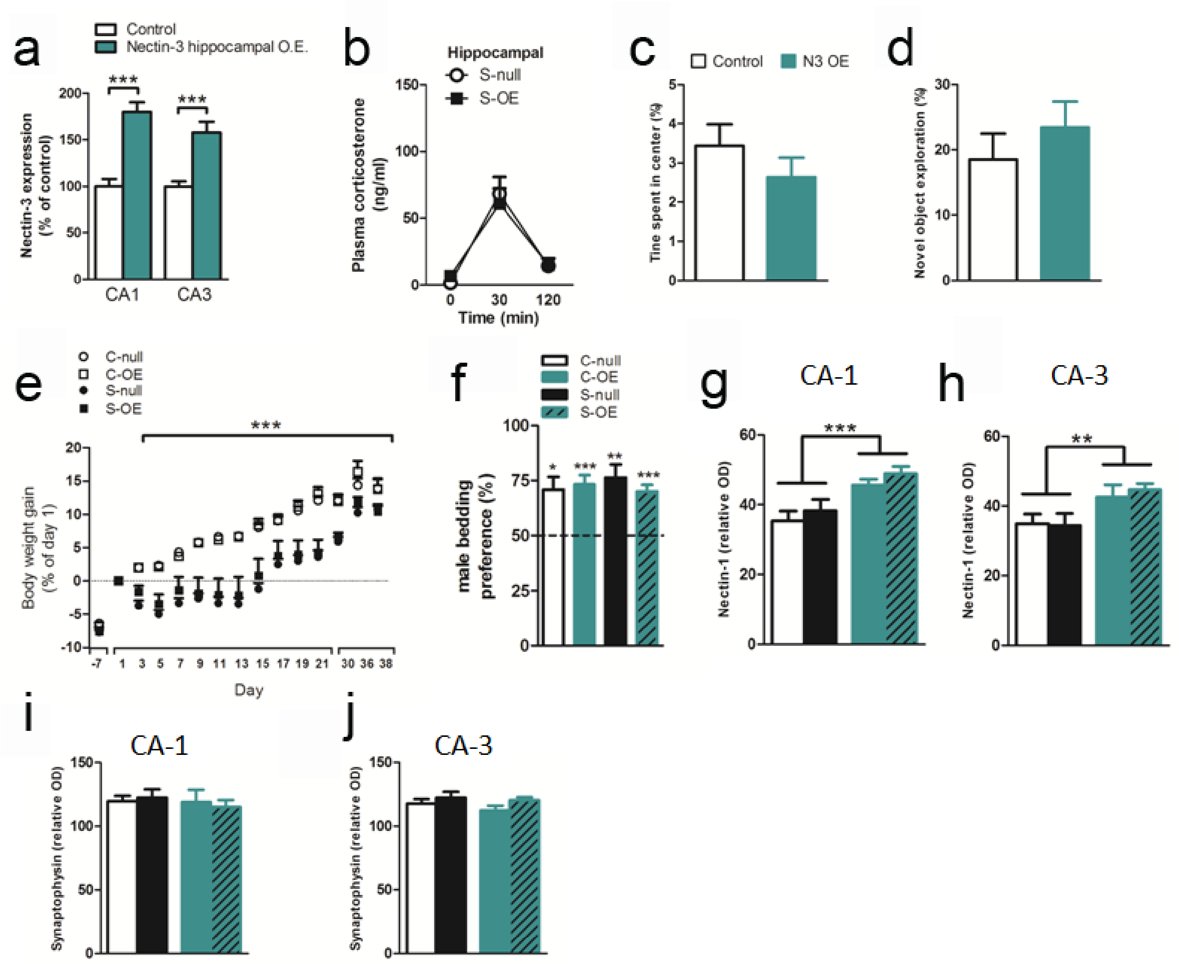

Supplementary Figure 3 (**a**) Nectin-3 expression was clearly increased in both CA1 and CA3 after hippocampal overexpression (OE) induced by viral transfection (CA1: t_{26}= 5.884, p<0.0001, CA3: t_{26}= 4.086, p= 0.0004 control n= 12 and nectin-3 OE n= 16). (**b**) Restraint stress significantly increased plasma corticosterone levels at 30 min of restraint stress and these levels were reduced at 120 min, plasma corticosterone in nectin-3 OE-animals (S-OE) did not differed animals treated with empty vectors (S-null) (n= 7/group). (**c-d**) Anxiety-like behavior as indicated by time spent in the center of the open field or exploration of a novel object were not affected 4 weeks after the induction of nectin-3 overexpression in the hippocampus (**c**: t_{24}= 1.079, p= 0.2912 n=13/group; **d**: t_{30}= 0.8665, p= 0.3931 n=16/group). (**e**) Restraint stress (S) significantly decreased bodyweight gain as compared to controls (C) but this was not affected by nectin-3 overexpression (effects of stress: $F_{1,30}$= 123.7, p<0.0001). (**f**) Bedding preference was assessed the day after the 21d stress paradigm. All animals tested displayed a preference for soiled male bedding as compared to clean bedding indicating an absence of anosmia. We observed no effect of nectin-3 overexpression on bedding preference (one-sample t-test, C-null: t_5= 3.689, p= 0.0142, n= 6; C-OE: t_7= 5.637, p= 0.0008, n= 8; S-null: t_7= 4.392, p= 0.0032, n= 8 and S-OE: t_7= 6.665, p= 0.0003, n= 8, one-sample t-tests against chance). (**g-h**) Nectin-1 and (**i-j**) synaptophysin expression was assessed in CA1 (**g,i**) and CA3 (**h,j**) areas. AAV-induced Nectin-3 overexpression increased nectin-1 expression in both CA1 and CA3 fields (**g,h**): N3 main effect $F_{1,26}$= 17,64 p= 0.0003 and $F_{1,26}$= 10,21 p= 0.0036, p= 0.0253, n= 5-8/group respectively for CA1 and

CA3. The expression of the synaptic marker synaptophysin (**i,j**) was unchanged by either N3 or stress (n= 6-8/group). Data are expressed as average OD value ± SEM. **p<0.01, ***p<0.001.

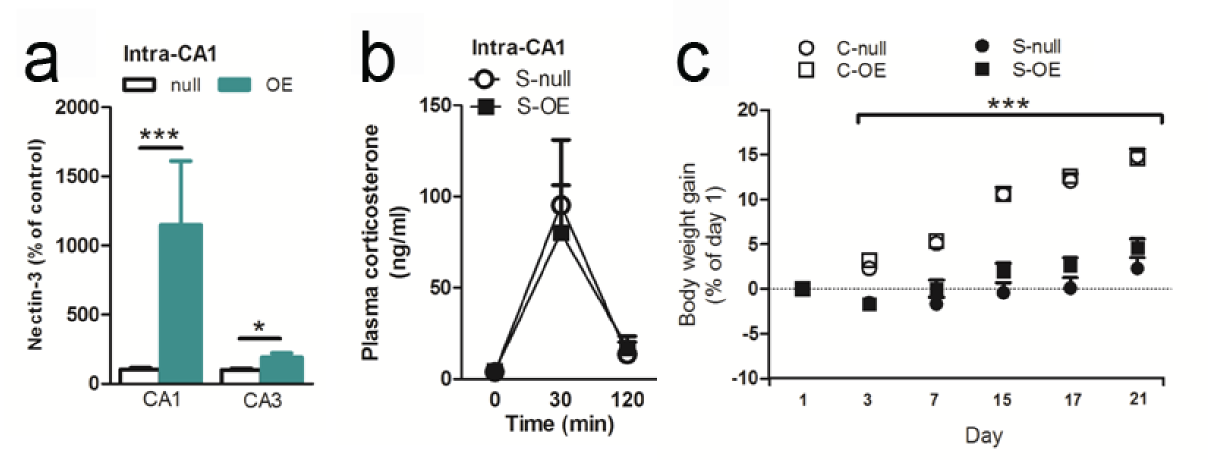

Supplementary Figure 4 (**a**) Viral overexpression of nectin-3 when injected intra-CA1 predominantly affects nectin-3 expression in the CA1 where local nectin-3 was strongly increased (U= 14, p<0.0001, CA1-null n= 16, CA1-OE n= 14); nectin-3 in the CA3 showed a modest increase (U= 45, p= 0.0476, CA1-null n= 12, CA1-OE n=14) (Mann-Whitney test) (n= 14-16/group).(**b**) Restraint stress significantly increased plasma corticosterone levels at 30 min of restraint stress and these levels were reduced at 120 min, plasma corticosterone levels did not differ between animals treated with AAVs to overexpress nectin-3 and empty vectors (null) (stress-null n= 15, stress-OE n= 14). (**c**) Restraint stress (S) significantly decreased bodyweight gain as compared to controls (C) but this was not affected by inta-CA1 nectin-3 overexpression (effects of stress $F_{3,324}$= 191, p<0.0001, control-null n= 14, control-OE n= 16, stress-null n= 14, stress-OE n= 14). ***p<0.001.

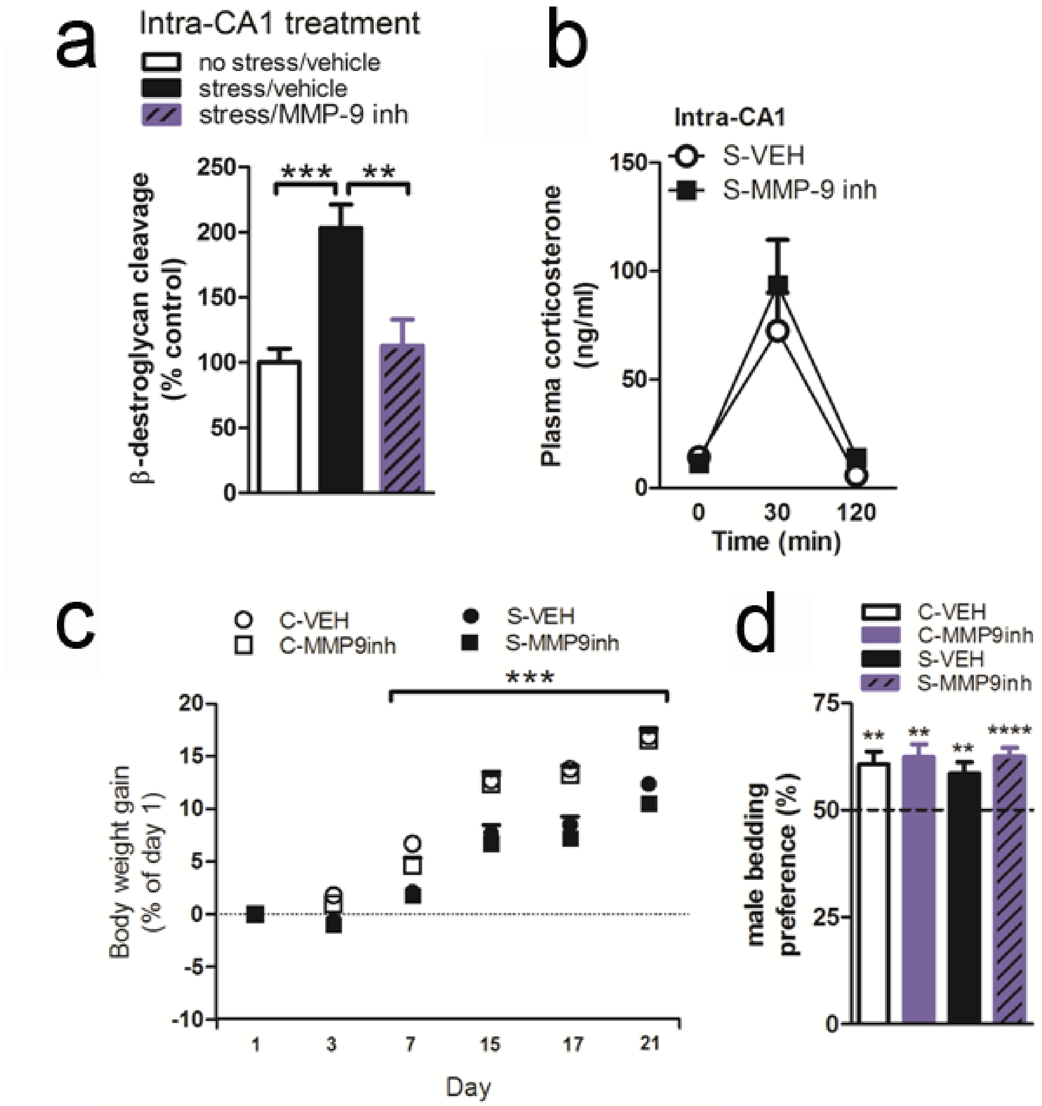

Supplementary Figure 5 (**a**) The effectiveness of the MMP-9 inhibitor was tested on its capacity to reduce β-destroglycan cleavage. Stress increased β-destroglycan cleavage and this could be prevented by infusion of a specific MMP-9 inhibitor ($F_{2,25}$= 14.15, p<0.0001, control vs. stress, t= 5.083, p<0.0001 and stress/vehicle vs. stress/mmp-9 inhibitor, t= 3.551, p<0.01, no stress/vehicle n= 11, stress/vehicle n= 10, stress/MMP-9 inh n= 5). (one-way ANOVA with bonferroni post hoc test). (**b**) Restraint stress significantly increased plasma corticosterone levels at 30 min of restraint stress and these levels were reduced at 120 min, plasma corticosterone levels did not differ between animals treated with the specific MMP-9 inhibitor or vehicle (n= 17/group). (**c**) Restraint stress (S) significantly decreased bodyweight gain as compared to controls (C) but this was not affected by MMP-9 inhibition (effects of stress, $F_{3,372}$= 69.94, p<0.0001, control-veh n= 14, control-MMP-9 inh n= 17, stress-veh n= 14, stress-MMP-9 in n= 18). (**d**) Bedding preference was assessed the day after the 21d stress paradigm. All animals tested displayed a preference for soiled male bedding as compared to clean bedding indicating an absence of anosmia. We observed no effect of MMP-9 inhibition on bedding preference (C-VEH: t_{12}= 3.510, p= 0.0043, n= 13; C-MMP9inh: t_{11}= 4, p= 0.0021, n= 12; S-VEH: t_{14}= 3.299, p= 0.0053, n= 15 and S-MMP-9inh: t_{15}= 6.020, p<0.0001, n= 16) (one-sample t-tests against chance). **p<0.01, ***p<0.001.

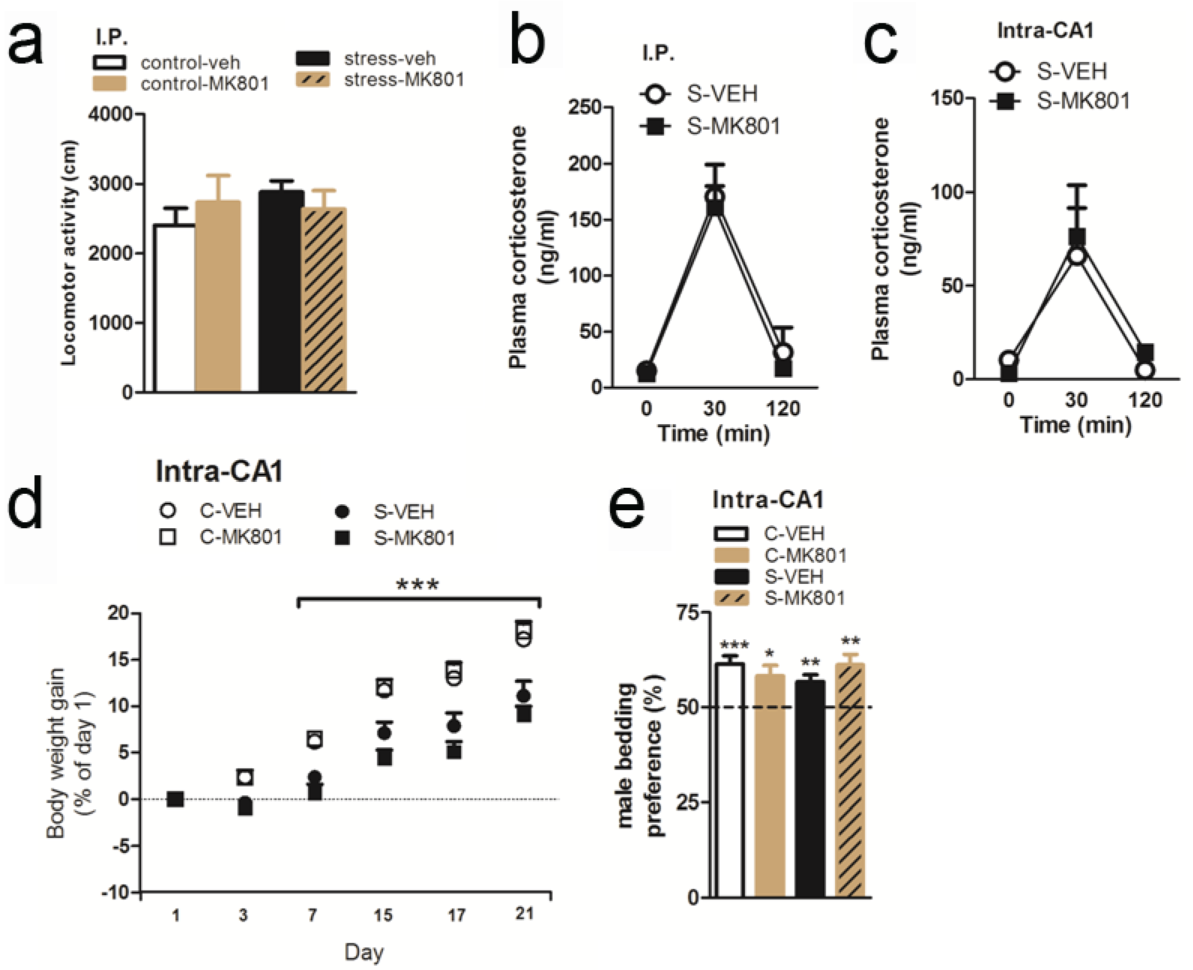

Supplementary Figure 6. (**a**) Intraperitoneal (i.p.) injection of the NMDA-antagonist MK-801 did not affect locomotor activity ($F_{1,35}$= 0.07, p= 0.8699, n= 10/group but n= 9 for C-MK) (2 way ANOVA). (**b-c**) Restraint stress (S) significantly increased plasma corticosterone levels at 30 min of restraint stress and these levels were reduced at 120 min, plasma corticosterone levels did not differ between animals treated with MK-801 or vehicle (n= 8-9/group). (**d**) Restraint stress significantly decreased bodyweight gain as compared to controls (C) but this was not affected by MK-801 treatment (effects of stress, $F_{3,354}$= 61.23, p<0.0001, C-VEH n= 12, C-MK n= 15, S-VEH n= 17, S-MK n= 19). (**e**) Bedding preference was assessed the day after the 21d stress paradigm. All animals tested displayed a preference for soiled male bedding as compared to clean bedding indicating an absence of anosmia. We observed no effect of MK-801 treatment on bedding preference (C-VEH: t_{12}= 5.317, p= 0.0002, n= 13; C-MK-801: t_{11}= 3.021, p= 0.0116, n= 12; S-VEH: t_{15}= 3.616, p= 0.0025, n= 16 and S-MK-801: t_{17}= 3.939, p= 0.0011, n= 18) (one-sample t-tests against chance). *p<0.05, **p<0.01, ***p<0.001.

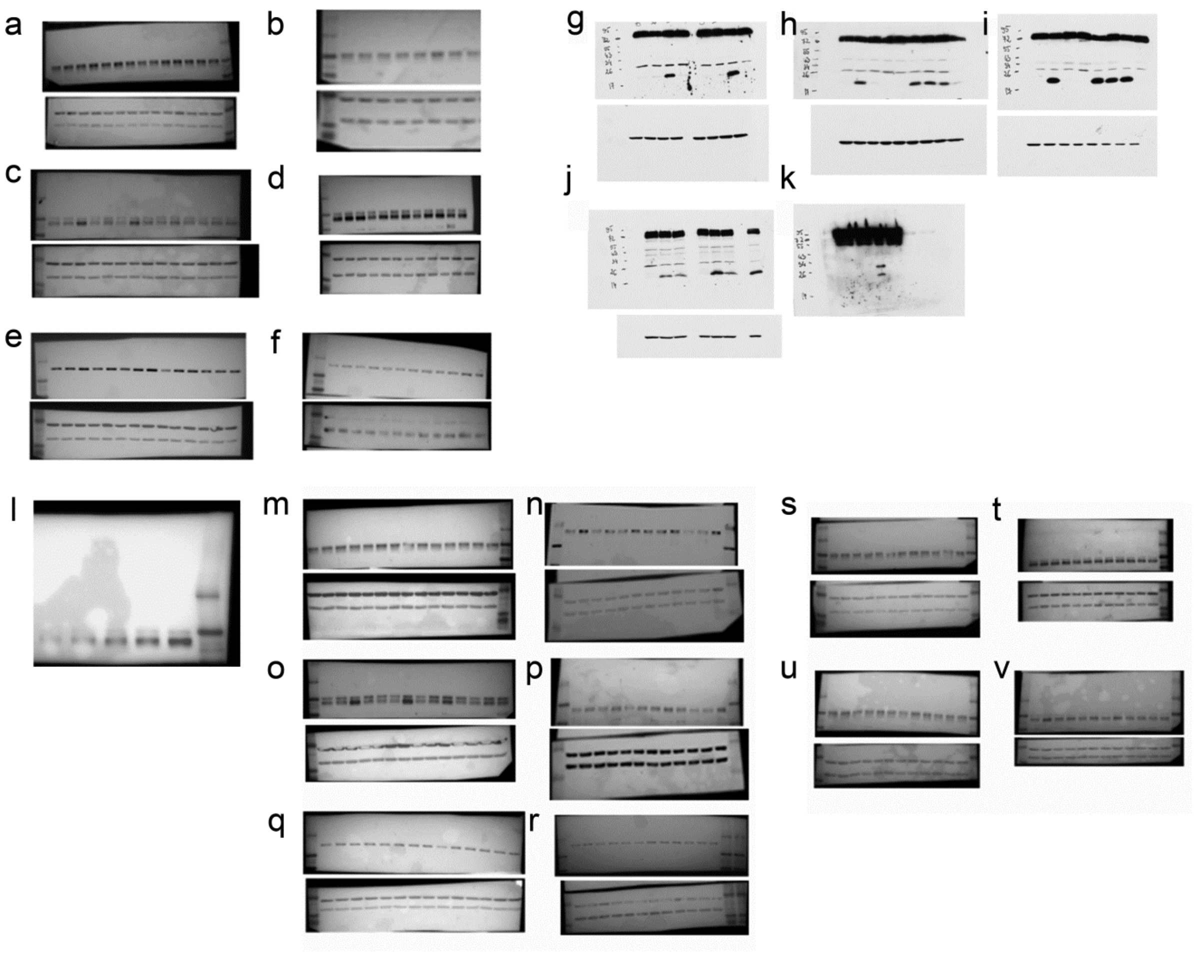

Supplementary Figure 7 All non-cropped Western blots are shown for the representative blots in the figures. Blots shown for figure 1d (**a-f**): the blots are shown in pairs; top part shows nectin-1 (**a-b**), nectin-3 (**c-d**), N-cadherin (**e-f**) while the lower band in the lower parts show GAPDH along with the molecular markers. The blots are shown for the 1d of restraint stress condition (panels **a**, **c** and **e**) and for the 21d restraint stress condition (panels **b,d,f**), Blots shown for figure 4 (**g-k**); the blots in figure 4a-e correspond to panels **g-k** respectively. The top panels display nectin-3 protein expression while the lower parts show actin-expression. Panel **l** shows the full blot for linear expression of nectin-3 as shown in supplementary figure 1; loaded are 3.75, 5, 7.5, 12 and 15 μg of protein in lanes 1-5 respectively, lane 6 shows the molecular marker. Panels **m-r** show the corresponding non-cropped blots as shown in supplementary figure 2d: the blots are shown in pairs; top part shows nectin-1 (**m-n**), nectin-3 (**o-p**), N-cadherin (**q-r**) while the lower band in the lower parts show GAPDH along with the molecular markers. The blots are shown for the 1d of restraint stress condition (panels **m**, **o** and **q**) and for the 21d restraint stress condition (panels **n,p,r**), Panels **s-v** correspond to the representative blots shown in supplementary figure 2e-h respectively. The blots are shown in pairs; top blots show either nectin-1 (**s** and **u**) or nectin-3 (**t** and **v**) expression while the lower blots display GAPDH expression.

Supplementary Methods

Elevated plus maze

Anxiety-related behaviors were evaluated using the elevated plus maze test (EPM). The EPM consists of two opposing open arms (45 x 10 cm) and two enclosed arms (45 x 10 x 38 cm) that emanate from a central platform (10 x 10 cm), at a 50 cm elevation. Light was adjusted to 10–12 lx in the center. The rats were placed individually in the center of the EPM and allowed to explore the maze for 5 min. The behavior was video recorded and analyzed using a computerized tracking system (Ethovision 3.1.16, Noldus IT, The Netherlands). The percentage of time spent in the open arms was taken as an indication for anxiety-like behavior.

Open field and novel object reactivity tests

The exploratory activity was assessed in the open field test (OF), which involved placing the animals in a circular open arena (100 cm ∅, 32-cm high). For the analysis, the floor was divided into three virtual concentric areas, with a center zone in the middle of the arena (20 cm ∅), an interior zone (60 cm ∅), and an exterior zone made up of the remaining area along the sidewalls. At the start of the test, the animals were placed in the center of the arena, and their behavior was monitored for 10 min. The distance moved (cm) and time spent (s) in each zone were analyzed using the video tracking system.

The three-chambered apparatus

The three-chambered apparatus consisted of a center- (20 × 35 × 35 cm), left- and right compartment (30 × 35 × 35 cm) made from gray opaque polycarbonate. The two lateral chambers contained a transparent Plexiglas cylinder (15 cm ∅ and 35 cm high) that was fixed to the floor of the apparatus. Cylinders contained either an unfamiliar male juvenile (J1, postnatal day 28 ± 2) or an unfamiliar object (yellow and blue empty container). The cylinders exhibited regular 0.5 cm holes that allowed olfactory and nose-to-nose contact. The dividing walls had retractable partitions that allowed access into each chamber. Light was maintained at 2-3 lx with homogeneous illumination. After a 2-min habituation period in the middle chamber, the retractable doors were removed, and the experimental rat was allowed to explore the entire apparatus for 5 min. The time spent sniffing either the juvenile (social target) or the novel object (inanimate target) was manually scored from videotapes by an experimenter blinded to the treatment groups. The time that the rats spent rearing with the forepaws on the cylinder was considered to be an exploration of the environment and was therefore excluded from the calculation.
